# Improving the breeding capabilities of short-term estrus synchronized Ossimi sheep using pregnant mare serum gonadotropin loaded chitosan-nanoparticles

**DOI:** 10.3389/fvets.2025.1607674

**Published:** 2025-06-03

**Authors:** Ahmed M. Shehabeldin, Maha S. Salama, Mohamed E. A. Omar, Liza A. AbdEl-Rafaa, Mohey A. Ashour, Mahmoud A. E. Hassan, Abdelghany A. El-Shereif, Mohamed Abdelmegeid, Mustafa Shukry, Ahmed A. Elolimy

**Affiliations:** ^1^Animal Production Research Institute (APRI), Agricultural Research Center (ARC), Giza, Egypt; ^2^Animal Reproduction Research Institute (ARRI), Agricultural Research Center (ARC), Giza, Egypt; ^3^Riwina Animal Production Farm, Agricultural Research Center (ARC), Ministry of Agriculture, Kafr El Sheikh, Egypt; ^4^Animal and Fish Production Department, Faculty of Agriculture (El-Shatby), Alexandria University, Alexandria, Egypt; ^5^Department of Animal Medicine, Faculty of Veterinary Medicine, Kafrelsheikh University, Kafr El Sheikh, Egypt; ^6^College of Veterinary Medicine, University of Al Dhaid, Sharjah, United Arab Emirates; ^7^Physiology Department, Faculty of Veterinary Medicine, Kafrelsheikh University, Kafr El Sheikh, Egypt; ^8^Department of Integrative Agriculture, College of Agriculture and Veterinary Medicine, United Arab Emirates University, Al Ain, United Arab Emirates

**Keywords:** estrus synchronization, PMSG, PMSG-CsNPs, Ossimi ewes, reproductive parameters

## Abstract

**Introduction:**

The present study evaluated the efficacy of pregnant mare serum gonadotropin (PMSG) and PMSG encapsulated in chitosan-tripolyphosphate nanoparticles in enhancing reproductive performance in short-term progesterone estrus-synchronized Ossimi ewes.

**Methods:**

Seventy-five healthy ewes were randomly assigned to three groups (*n* = 25 per group). Group 1 (representing current standard practice) received 25 mg progesterone acetate for 7 days, 600 IU PMSG on day 6, and 250 μg prostaglandin F_2α_ (PGF_2α_) on day 7. Group 2 followed the same regimen as Group 1, except for administering 300 IU of PMSG-loaded chitosan-tripolyphosphate nanoparticles on day 6, followed by an intramuscular injection of 250 μg PGF_2α_ on day 7. Group 3 received 150 IU of PMSG-loaded chitosan-tripolyphosphate nanoparticles on day 6 and 250 μg PGF_2α_ on day 7. Estrus detection occurred between days 7 and 11, with a gonadotropin-releasing hormone (GnRH) injection at breeding.

**Results and discussion:**

Group 1 had a significantly shorter onset of estrus (54.40 ± 4.50 h; *p* < 0.05) compared to Group 2 (71.60 ± 0.51 h) and Group 3 (72.20 ± 4.81 h). Pregnancy and lambing rates were highest in Group 2 (100%; *p* < 0.05), and Group 2 produced more fetuses (40) than Group 1 (30) and Group 3 (25). Fecundity was also highest in Group 2 (160%; *p* < 0.05). Follicular diameter was greater in Group 2 on day 9, although the number of large follicles was similar across groups. The number of corpora lutea significantly increased on day 7 compared to day 0 in all groups. Progesterone levels peaked on day 7 and declined by day 9 across all groups. These results suggest that administering 300 IU of PMSG encapsulated in chitosan-tripolyphosphate nanoparticles can enhance reproductive performance more effectively than conventional PMSG, offering a promising strategy to improve fertility in short-term progesterone-synchronized ewes.

## 1 Introduction

Ossimi sheep are one of the main Egyptian sheep breeds (Ossimi, Rahmani, Saidi, and Barki) known for their high fertility and ability to cycle throughout the year, potentially giving birth more than once annually (1). Many studies have shown that Ossimi sheep excel in accelerated lambing programs (2), with a 95% success rate for September mating. The twinning rate among Ossimi sheep is moderate at 14%, influenced by breeding season and geographical location factors (3), with an ovulation rate of 1.2–1.37 per ewe.

According to Garoussi et al. (4), estrus synchronization is essential for enhancing sheep reproduction. Recent research has focused on the duration of these synchronizations, which can vary from short-term to long-term procedures lasting 7 to 14 days (5). These methods are equally effective in inducing viable estrus and ovulation in breeding and non-breeding seasons (6). Various techniques have been developed for synchronizing ewes’ estrous cycles, such as the administration of intramuscular injections of progesterone acetate in oil every other day for 6 or 12 days, along with an infusion of eCG on the last injection day, as described in studies by Almadaly et al. (7).

Pregnant mare serum gonadotropin (PMSG), equine chorionic gonadotropin (eCG), is a hormone in pregnant mares. It can enhance reproductive success by increasing the likelihood of superovulation and pregnancy while reducing inconsistencies in estrus presentation (8). PMSG is a molecule with biological functions similar to luteinizing hormone (LH) and follicle-stimulating hormone (FSH) (9). PMSG has a longer half-life than pituitary and placental gonadotropin hormones, lasting 40–125 h (10), whereas FSH and LH only endure for 3–5 h or even as little as 30 min (11). Various methods of utilizing PMSG for superovulation purposes include increasing the quantity of preantral follicles, preserving early-stage atresia follicles to facilitate growth and ovulation, accelerating the development of small follicles into larger ones, and decreasing the proportion of antral follicles undergoing atresia by stimulating the growth of larger or smaller antral follicles (12). Nanoparticles are currently becoming an important component in various industries. Many processes and applications have become more effective, economical, and time-saving due to nanoparticles. While polymers with distinct characteristics are commonly used, natural polymers possess important properties that make them suitable for nanoparticle production. Different techniques, like ionotropic gelation, micelles, and emulsification, have been effectively used to create chitosan nanoparticles (CsNPs) that can carry various substances. Chitosan nanoparticles have a wide range of applications, including improving bioavailability, controlling drug release, increasing cellular uptake, and targeting cancer cells, stabilizing proteins, and enhancing the effectiveness of antimicrobial agents (13). Nanoparticles are microscopic particles, with a significant portion of their atoms on the surface (14). Recent studies by Esquivel et al. (15) have shown that advancements in nano-drug delivery methods can extend the lifespan of medication, aid in its movement through the body’s barriers, and ensure precise delivery to the target site. These modifications can enhance drug absorption at a cellular level, potentially allowing for lower dosage requirements. Consequently, using nano-drug systems in hormonal treatments could have significant implications for various processes in animal reproduction (16). As a result, we propose that injecting PMSG-loaded chitosan-tripolyphosphate (TPP) nanoparticles may serve as an alternative to PMSG in estrus synchronization protocols by enhancing follicular growth before ovulation. Therefore, this study was conducted first with PMSG-loaded chitosan-TPP nanoparticles to investigate whether the intramuscular injection of PMSG-loaded chitosan-TPP nanoparticles could improve the reproductive performance of short-term progesterone-synchronized ewes.

## 2 Materials and methods

### 2.1 Ethical statement

The animal study was examined and approved by the Animal Ethics Committee of the Faculty of Veterinary Medicine, Kafrelsheikh University, Egypt (KFS-IACUC/125/2023). The studies were conducted in accordance with the local legislation and institutional requirements.

### 2.2 Animals

Seventy-five healthy Egyptian Ossimi ewes, aged 4–6 years, with 2–3 parities, and weighing 45–55 kg, were selected based on a good body condition score of 2.5–3.25 based upon a scale of 1–5 (17). All animals used in this study were obtained from the governmental Sakha Animal Production Research Station. The animals were housed and managed according to the station’s standard protocols, ensuring consistency in husbandry and care; the research was conducted during the breeding season at the Sakha Animal Production Research Station, Agricultural Research Center, Kafrelsheikh, Egypt (Latitude 31° 06’ N, Longitude 30° 56′E), in September–October 2023. The sheep were housed in a semi-open yard, fed a combination of concentrate feed and roughages following NRC (18) guidelines, and had unlimited access to fresh water.

### 2.3 Creation and evaluation of hormone-conjugated chitosan nanoparticles

The ionic gelation process, as described by Gallab et al. (19), was used to create bare chitosan-tripolyphosphate nanoparticles. Highly purified chitosan (Alpha Chemika®, Mumbai, India) with a deacetylation percentage of over 85% and a low molecular weight was utilized. Additionally, sodium tripolyphosphate (TPP, Thermo Fisher®, GmbH, Germany) was used for the formulation of the polymeric nanocarrier. Initially, bare chitosan-tripolyphosphate nanoparticles were formed by slowly adding the TPP aqueous solution (0.1 gm/dL) into the chitosan solution in an aqueous acid solution (0.1%, w/v) at a 1:2 ratio, respectively. Pregnant Mare Serum Gonadotropin hormone solution (1 mL Folligon®, a white freeze-dried crystalline powder in vials of 1.000 IU together with solvent for reconstitution) was added drop by drop to the pre-structured CsNPs in a 1:1 ratio to produce 100 IU PMSG-loaded chitosan nanoparticles (PMSG-CsNPs). The mixture was pH-adjusted to 6.5, gently stirred for 60 min at 300 × g, and then incubated overnight to ensure proper hormonal adsorption on the nanoparticles’ surface. To measure the hormonal loading efficiency (LE%) of CsNPs, a centrifugation process at 1,200 × g for 20 min was used for CsNPs and PMSG-CsNPs solutions to separate the nanoparticles from the aqueous medium. The quantity of free hormone in the supernatant of the PMSG-CsNPs solution was measured using a UV spectrophotometer (Optizen Pop, Mecasys Co., Ltd., Korea) at a wavelength of 280 nm (20), with the loading efficiency percentage calculated as (Total hormone − Free hormone in supernatant)/Total hormone × 100.

For the assessment of nanoparticle characteristics, a Zeta sizer (Malvern Instruments, United Kingdom) was used with dynamic light scattering (DLS) to measure particle size, size distribution (polydispersity index; PdI), and zeta potential for both CsNPs and PMSG-CsNPs. The samples were measured in triplicate, and the average values of each parameter (± SEM) were determined.

### 2.4 Experimental design

**Figure 1:**
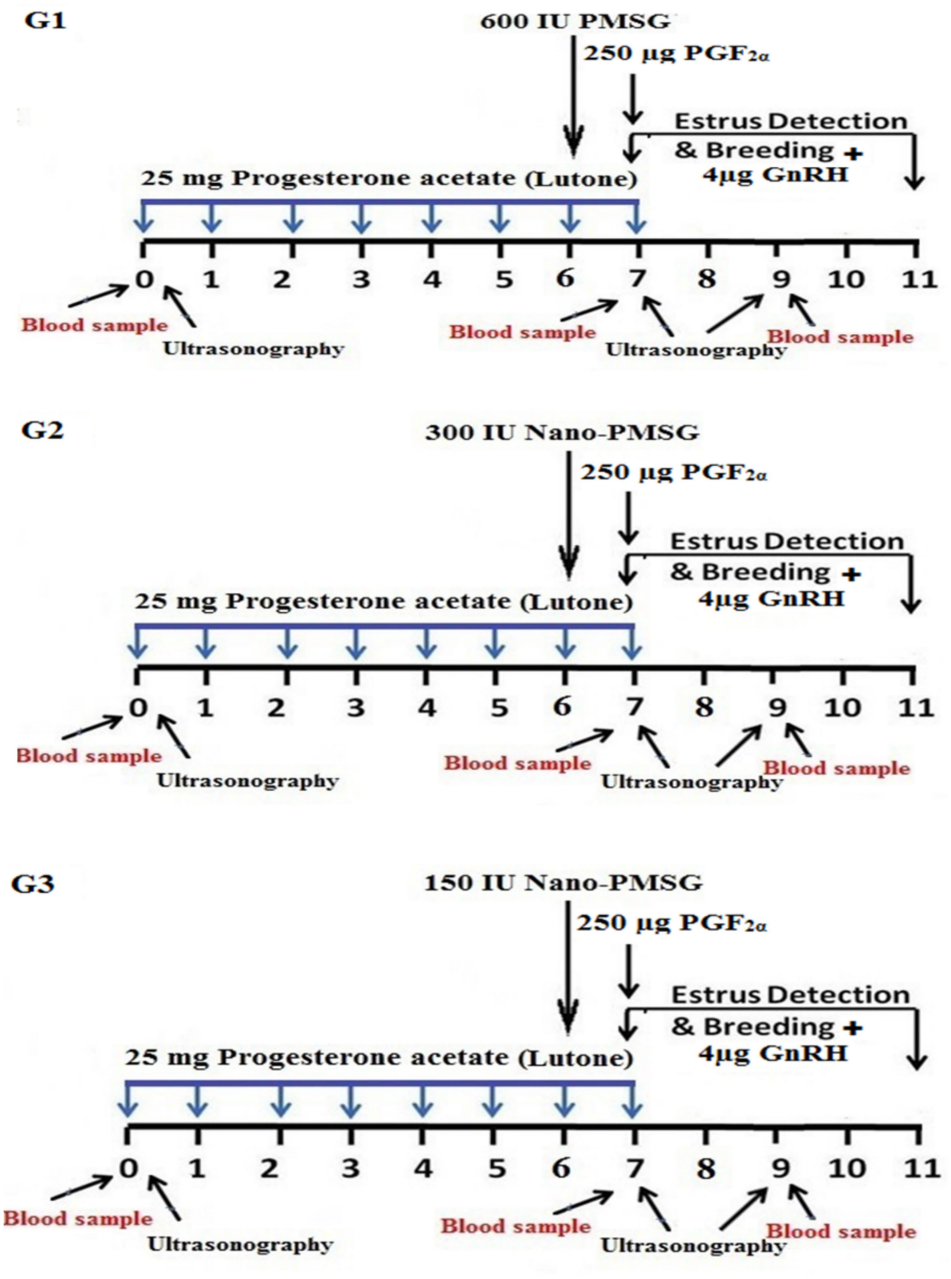
Diagram illustrating estrus synchronization procedures. Short-term progesterone estrus synchronization protocols using PMSG and PMSG-loaded chitosan nanoparticles were implemented. In Group 1, ewes were synchronized with 1 mL of Lutone (25 mg of progesterone acetate in oil) daily for 7 consecutive days. On the 6th day, they received 600 IU of PMSG (Folligon®), followed by 1 mL of Estrumate (250 μg of PGF_2α_) on the 7th day. Estrus detection was conducted with an intramuscular injection of 1 mL of Receptal (4 μg of GnRH) at breeding. In Group 2, the treatment regimen was the same as Group 1, except that on the 6th day, ewes received 300 IU of PMSG-loaded chitosan nanoparticles. Group 3 followed a treatment protocol similar to Groups 1 and 2, except for administering 150 IU of PMSG-loaded chitosan nanoparticles on the 6th day.

All experiments began on a randomly selected day, designated as day 0. Ewes were randomly assigned to three homogeneous groups based on body condition score, age, and parity. Each group was assigned a reference color and treated according to the experimental design outlined in Figure 1.

Group 1 (G1), serving as the control group, received the conventional short-term synchronization protocol; each ewe received 25 mg of progesterone acetate in oil [1 mL Lutone (Misr, Egypt)] intramuscularly daily for 7 consecutive days. On the 6th day, they received an intramuscular injection of 600 IU of PMSG [3 mL Folligon® (MSD, Intervet International B.V., Boxmeer, The Netherlands)], followed by 250 μg of PGF_2α_ [1 mL Estrumate (PGF2alpha analog, cloprostenol sodium, Coopers Animal Health Ltd., Berkhamsted, England)] on the 7th day. Estrus detection was conducted from the last day to the 11th day, with an intramuscular injection of 4 μg of GnRH [1 mL Receptal (GnRH analog, buserelin acetate, Intervet International, Netherlands)] at breeding, as illustrated in Figure 1.

Group 2 (G2), the treatment regimen was identical to that of group 1, except for the type and dose of PMSG (300 IU Nano-PMSG) administered on the 6th day, as depicted in Figure 1.

Group 3 (G3), the treatment protocol mirrored that of groups 1 and 2, except for PMSG (150 IU Nano-PMSG) administered on the 6th day, as indicated in Figure 1.

### 2.5 Estrus characteristics and estrus rate

After synchronization was completed on the 7th day, estrus detection was carried out twice daily (every 12 h) for 3 h each time. Estrus symptoms were monitored using vasectomized rams for 4 days. Once estrus was confirmed, three fertile rams (aged 4–5 years) were used to breed 75 ewes (1 ram per 25 ewes). The end of the estrus was confirmed when the ewe refused to be mounted by the ram, and this period was observed every 4 h for 1 h. The effectiveness of the three protocols was evaluated based on the onset and duration of estrus (hours) as well as the estrus rate (number of ewes in estrus divided by the number of treated ewes, multiplied by 100).

### 2.6 Reproductive efficiency

Ultrasound scanning was used to diagnose pregnancy in ewes using an Esaote transrectal linear probe [a real-time B-mode veterinary ultrasound device (Esaote Pie Medical Aquila Pro Vet with 6.0/8.0 MHz LA Rectal Veterinary, Esaote, Italy)], from day 45 to day 50 post-natural breeding, followed by confirmation at 150 days to avoid early embryonic losses. Positive indicators of pregnancy included the detection of the embryonic vesicle, embryo proper, or placentomes. Reproductive efficiency parameters were determined by calculating the pregnancy rate (Number of pregnant ewes divided by the number of mated ewes), lambing rate (Number of ewes giving live lambs divided by the number of mated ewes), prolificacy rate (Number of fetuses divided by the number of ewes lambing multiplied by 100), fecundity rate (Number of fetuses divided by the number of inseminated ewes multiplied by 100), twinning rate (%), and the total number of fetuses.

### 2.7 Ovarian ultrasonography

On days 0, 7, and 9 of treatment, the ovaries of each ewe were observed using transrectal scanning [a real-time B-mode veterinary ultrasound device (Esaote Pie Medical Aquila Pro Vet with 6.0/8.0 MHz LA Rectal Veterinary, Esaote, Italy)]. Each ovary underwent a complete pole-to-pole scan, with pictures taken and frozen of various ovarian portions (21). The total number and diameter of follicles (small (2–2.9 mm), medium (3–5 mm), and large (>5 mm)) within the ovaries were estimated, and the number of corpus luteum (CL) within the ovaries was also counted.

### 2.8 Progesterone assay

Blood samples were taken from 75 ewes on days 0, 7, and 9 from the beginning of the treatment. The blood was collected in tubes without anticoagulants and spun in a centrifuge at 1,500 × g for 15 min. The serum was frozen at −80°C for progesterone (P4) analysis. The Coat-A-Count Progesterone® radioimmunoassay kit (Diagnostic Products Corporation, Los Angeles, United States) was used according to the manufacturer’s instructions. Calibration standards ranged from 0.1 to 60 ng/mL, and the assay demonstrated a sensitivity of 0.09 ng/mL. The intra-assay and inter-assay coefficients of variation (CV) were 5.1% and 4.2%, respectively.

### 2.9 Statistical analysis

The data from this study were statistically analyzed using a one-way ANOVA to assess the onset and duration of estrus, prolificacy, fecundity, large follicular number, diameter, and CL number. Using the General Linear Model procedure, the Chi-square (χ^2^) test was utilized to analyze categorical data on estrus, pregnancy, lambing, and twinning rates (22). Duncan assessed Differences among means using the Range of Multiple Tests (23). Statistical analyses of P4 levels, follicular number, and diameters between and among days in different groups were determined by repeated measures ANOVA. A two-tailed t-test was used to determine the CL numbers of the same groups between days 0 and 7. All results were presented as means (± SEM), except for categorical data, which were presented as percentages. Differences were considered significant at *p* < 0.05. A correlation heatmap showing the multivariate relationships among estrus and reproductive parameters was generated in RStudio (R version 4.0.2).

## 3 Results

### 3.1 Exploring the physical and chemical characteristics of chitosan and chitosan nanoparticles loaded with PMSG

**Figure 2:**
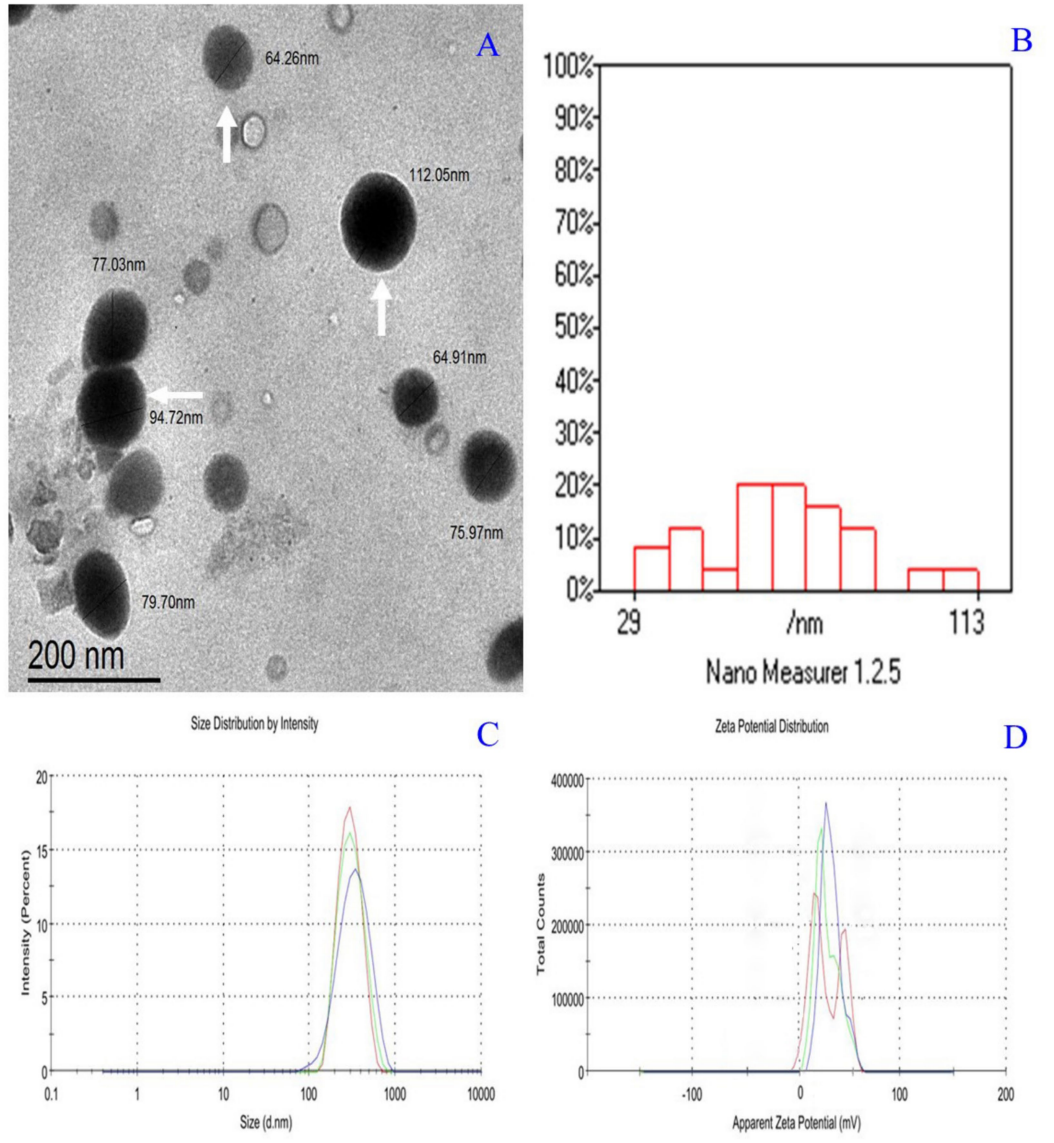
**(A)** Transmission electron microscope micrograph of PMSG nanoparticles showing nearly spherical (White arrows). **(B)** Histogram size distribution 29:113 nm. **(C)** Zeta size distribution by the intensity of PMSG nanoparticles Z-Average (d.nm):298 nm and PdI: 0.138. **(D)** Zeta potential distribution of PMSG nanoparticles Zeta Potential (mV): +11.

The physicochemical characterization of CsNPs and PMSG-CsNPs reveals significant differences. CsNPs exhibited a particle size of 47.82 ± 5.37 nm, a PdI of 0.263 ± 0.04, and a zeta potential of +37.0 mV. In contrast, PMSG-CsNPs displayed an increased particle size of 80.91 ± 4.73 nm, a lower PdI of 0.138 ± 0.07, and a reduced zeta potential of +11.0 mV, indicating successful PMSG loading with a high loading efficiency of 94.15%. Transmission electron microscope images showed spherical nanoparticles with sizes ranging from 29 to 113 nm, consistent with the DLS size distribution. The zeta potential distribution confirmed a shift in surface charge upon PMSG encapsulation, highlighting the stability and successful modification of the nanoparticles as illustrated in Table 1 and Figure 2.

**Table 1:** Physicochemical properties and loading efficiency of chitosan nanoparticles (CsNPs) and PMSG-loaded chitosan nanoparticles (PMSG-CsNPs).

Parameters	Particle size (nm)	PdI	Zeta potential (mV)	Loading efficiency (%)
CsNPs	47.82 ± 5.37	0.263 ± 0.04	+37.0	–
PMSG-CsNPs	80.91 ± 4.73	0.138 ± 0.07	+11.0	94.15%

PdI, Polydispersity index.

### 3.2 Estrus characteristics and estrus rate

In the first group (600 IU PMSG), synchronization resulted in a significantly earlier onset of estrus (54.40 ± 4.50 h, *p* < 0.05) compared to G2 and G3 (71.60 ± 0.51 h and 72.20 ± 4.81 h, respectively). However, the duration of estrus showed no significant differences between the three groups. All animals in each group exhibited estrus symptoms, with a 100% estrus rate in all groups (Table 2).

**Table 2:** The estrus characteristics and estrus rate of treated ewes (mean ± SEM).

Groups/parameters	*N*	Estrus characteristics		Estrus rate (%)
		Onset of estrus (h)	Duration of estrus (h)	
G1	25	54.40 ± 4.50^b^	27.40 ± 0.93^a^	(25/25) 100^a^
G2	25	71.60 ± 0.51^a^	27.20 ± 0.86^a^	(25/25) 100^a^
G3	25	72.20 ± 4.81^a^	25.60 ± 0.51^a^	(25/25) 100^a^

*N*, Number of ewes. Means and values with different superscript letters (a, b) in the same column differ significantly (*p* < 0.05).

### 3.3 Reproductive performance

Fortunately, there were no abortions or fetal deaths in this investigation; the lambing rate for each procedure was similar to the pregnancy rate (Table 3). According to the findings in Table 3, there were significant differences (*p* < 0.05) in pregnancy and lambing rates between all groups. G2 had higher pregnancy and lambing rates (100%; *p* < 0.05) than G1 and G3, while G3 had the lowest rates (60%). There were no significant differences between all groups in the twin rate, with the highest rate in G3 (66.67%). The number of fetuses was higher in G2 (40) than in G3 (25). Prolificacy (%) showed no significant differences between all groups, but fecundity (%) had significantly higher differences (160; *p* < 0.05) in G2 compared to G1 and G3 (Table 3).

**Table 3:** The reproductive efficiency of treated ewes.

Groups/parameters	Pregnancy rate (%)	Lambing rate (%)	Twining rate (%)	Fetuses (*n*)	Prolificacy (%)	Fecundity (%)
G1	(20/25) 80^b^	80^b^	(10/20) 50^a^	30	150^a^	120^b^
G2	(25/25) 100^a^	100^a^	(15/25) 60^a^	40	160^a^	160^a^
G3	(15/25) 60^c^	60^c^	(10/15) 66.67^a^	25	167^a^	100^b^

The number of ewes in each group is 25. Values with different superscript letters in the same column differ significantly (*p* < 0.05). Prolificacy = number of fetuses/number of pregnant ewes × 100. Fecundity = number of fetuses/number of inseminated ewes × 100.

### 3.4 Ovarian structures

**Figure 3:**
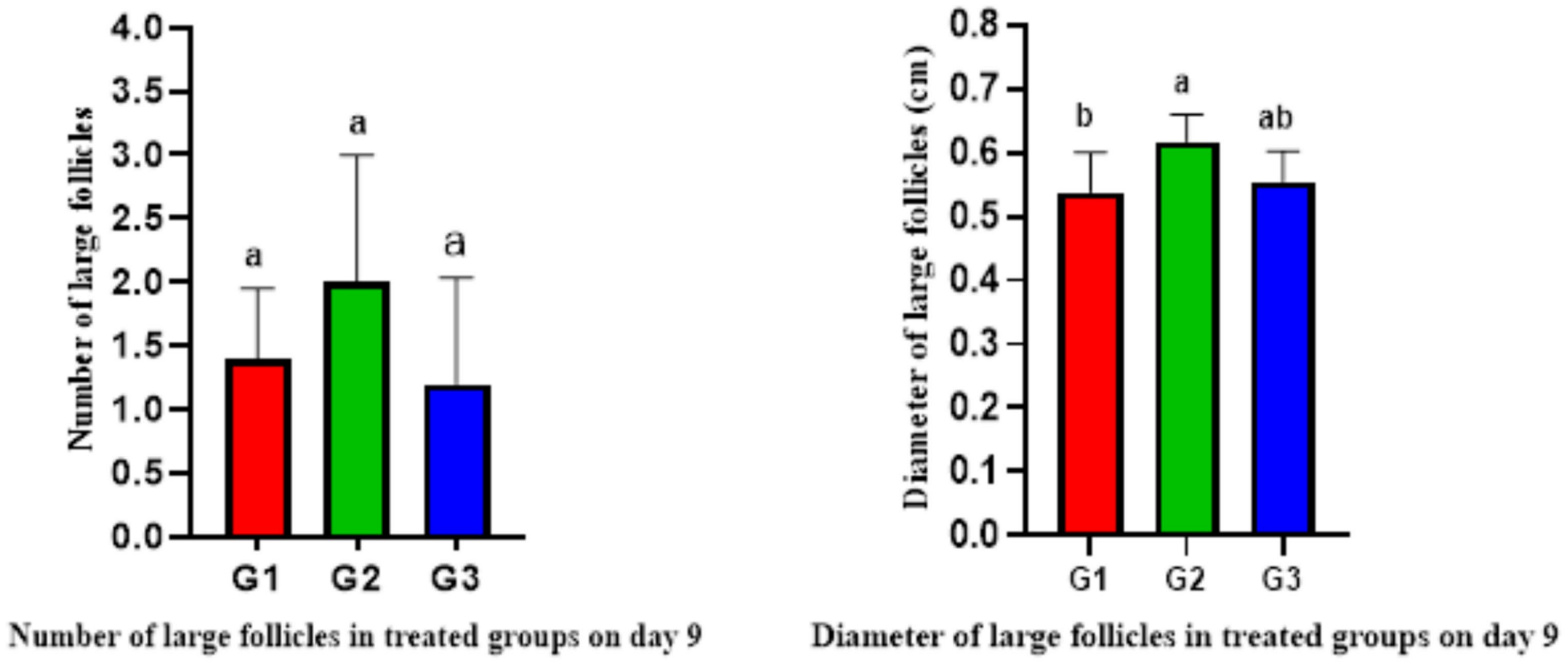
Number and diameter of large follicles in treated ewes on day 9 (mean ± SEM). Means with different superscripts (a, b) in different columns of the same figure indicate significant differences at *p* < 0.05.

The ultrasonography of ovarian structure on days 0 and 7 revealed no significant differences among all groups in total follicular numbers and diameter. However, on day 9, G1 had significantly fewer follicles than G2 and G3, while G2 had the largest follicular diameter (0.59 ± 0.02 cm) compared to G1 and G3 (Table 4). There were no significant differences in follicular numbers within the same groups on different days (0, 7, and 9). Regarding follicular diameter, day 9 showed significantly higher (*p* < 0.05) values than days 0 and 7 (Table 4). In terms of large follicles, only G1 had one large follicle on day 0, with no large follicles on day 7 in any group. On day 9, there were no significant differences in the number of large follicles among all groups, but the diameter of these follicles was higher in G2 compared to G1 (Figure 3).

**Table 4:** Number and diameter of follicles in treated ewes (mean ± SEM).

Parameters/groups	Days	G1	G2	G3
FN	0	3.00 ± 1.04^a^	2.60 ± 0.51^a^	2.60 ± 0.40^a^
	7	1.80 ± 0.20^a^	2.00 ± 0.45^a^	1.60 ± 0.25^a^
	9	1.60 ± 0.25^b^	3.80 ± 0.50^a^	3.00 ± 0.50^a^
FD (cm)	0	0.31 ± 0.06^a^	0.30 ± 0.02^a^	0.28 ± 0.02^a^
	7	0.26 ± 0.03^a^	0.30 ± 0.03^a^	0.26 ± 0.01^a^
	9	0.46 ± 0.02^b*^	0.59 ± 0.02^a*^	0.47 ± 0.03^b*^

The number of ewes in each group is 25; FN, Follicular number; FD, Follicular diameter. Means with different superscripts (a, b) in rows indicate significant differences at *p* < 0.05. Means marked with an asterisk in the same column for the same parameter also show significant differences (*p* < 0.05).

**Figure 4:**
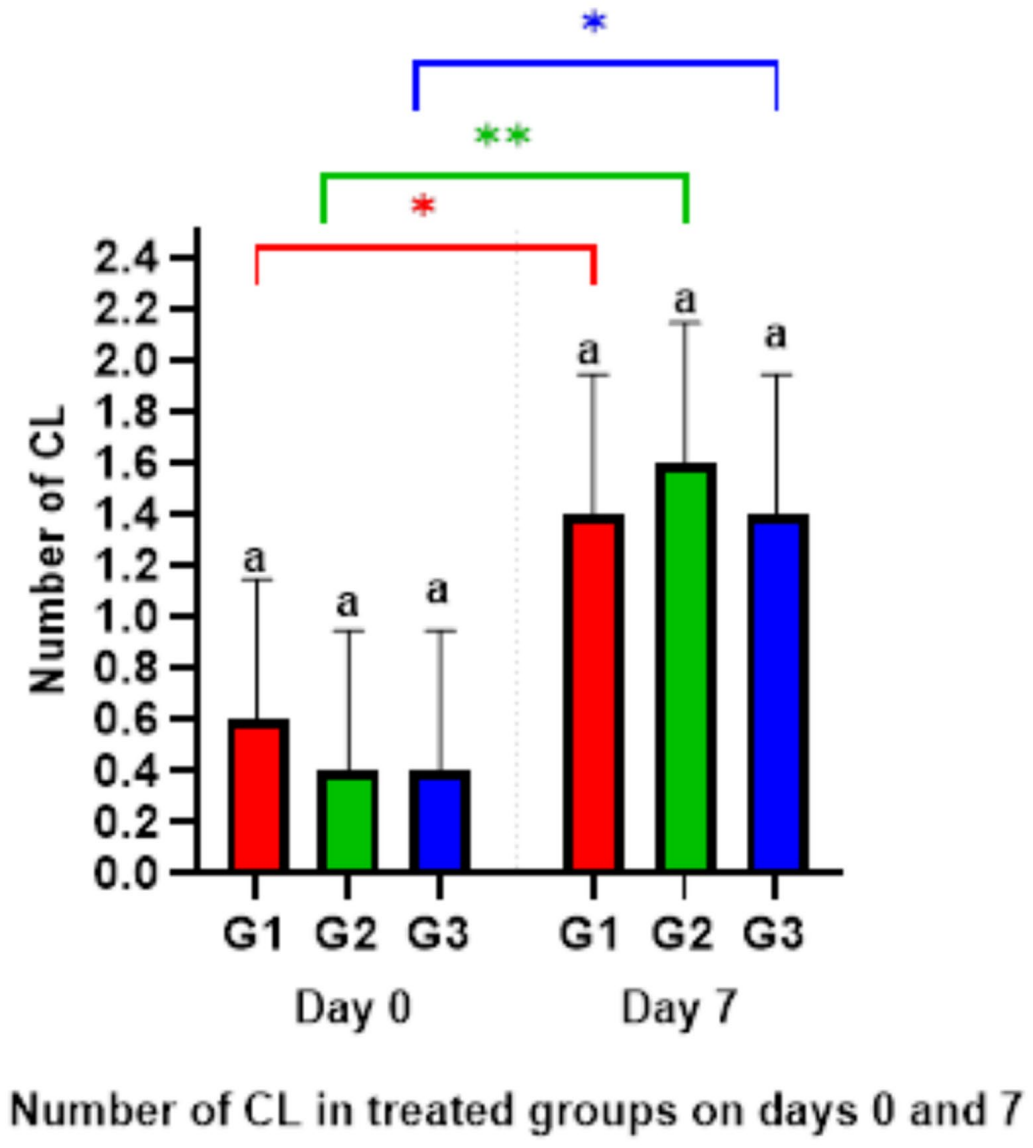
Number of corpus luteum on days 0 and 7. ^*^p < 0.05 G1 and G3 (Day 0) versus G1 and G3 (Day 7), ^**^p < 0.01 G2 (Day 0) vs. G2 (Day 7).

The ultrasonography of CL numbers showed no significant differences among all groups on days 0 and 7. However, the number of CL on day 7 significantly increased (*p* < 0.05) compared to the ipsilateral groups on day 0 (Figure 4). Meanwhile, there were no CL presents on day 9 in any group.

### 3.5 Progesterone profile

When comparing P4 concentrations among all groups on days 0, 7, and 9 separately, there were no statistically significant differences between the groups on different days (Table 5). However, when looking at P4 concentrations within each day, all groups on day 7 showed significantly higher differences (*p* < 0.05) compared to all groups on day 9, as well as G2 and G3 on day 0 (Table 5).

**Table 5:** The concentration of P4 (ng/ml) in treated ewes (mean ± SEM).

Days/groups	G1	G2	G3
0 d	1.37 ± 0.31^a*^	1.31 ± 0.37^a^	1.11 ± 0.38^a^
7 d	2.51 ± 0.51^a*^	3.52 ± 0.44^a*^	2.75 ± 0.53^a*^
9 d	0.77 ± 0.05^a^	0.66 ± 0.04^a^	0.81 ± 0.06^a^

The number of ewes in each group is 25. Means with different superscripts in the row indicate significant differences at *p* < 0.05. Means marked with asterisks in the same column show significant differences (*p* < 0.05).

### 3.6 Comparative analysis across groups

**Figure 5:**
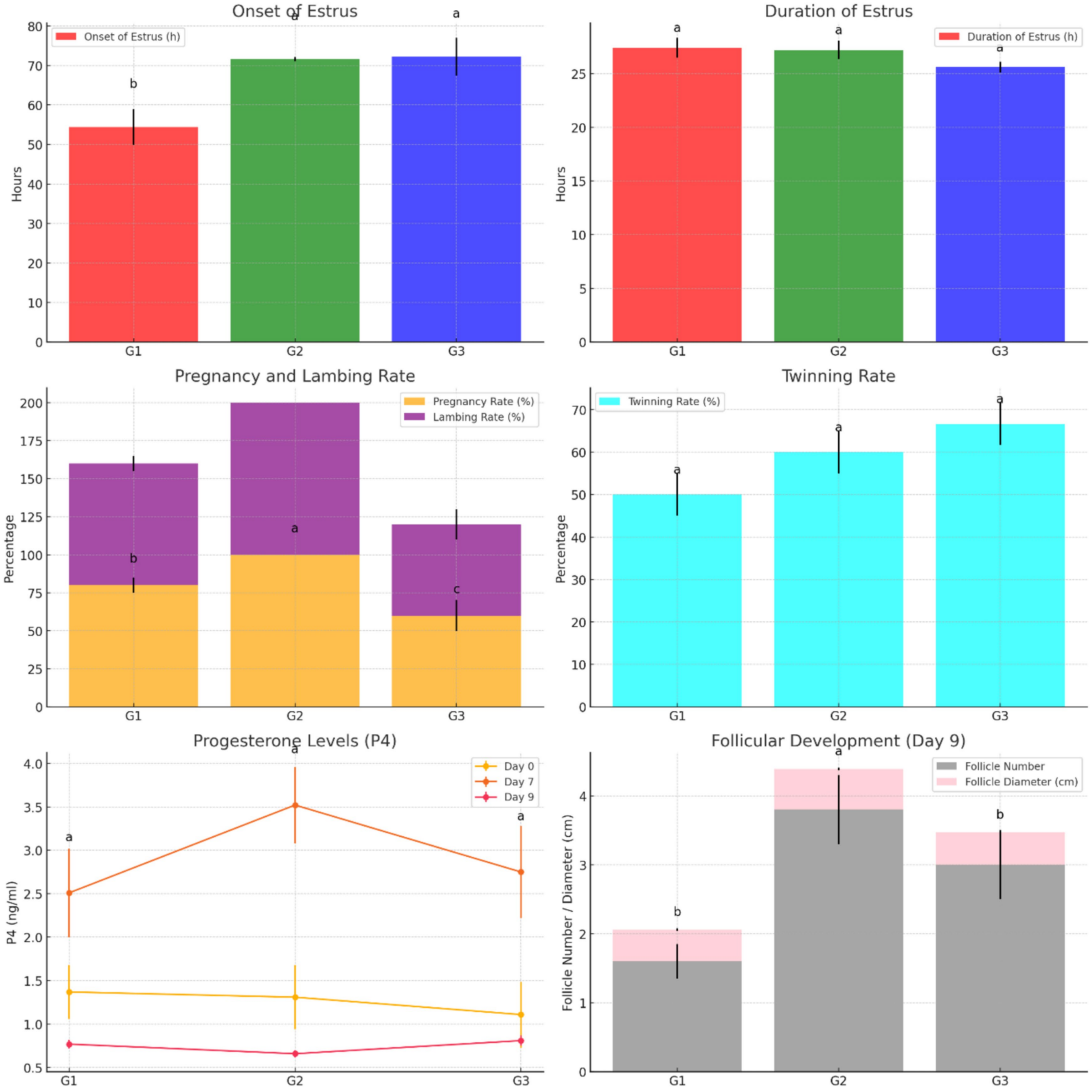
Comparison of estrus characteristics, reproductive efficiency, progesterone levels, and follicular development across treatment groups (G1, G2, G3).

**Figure 6:**
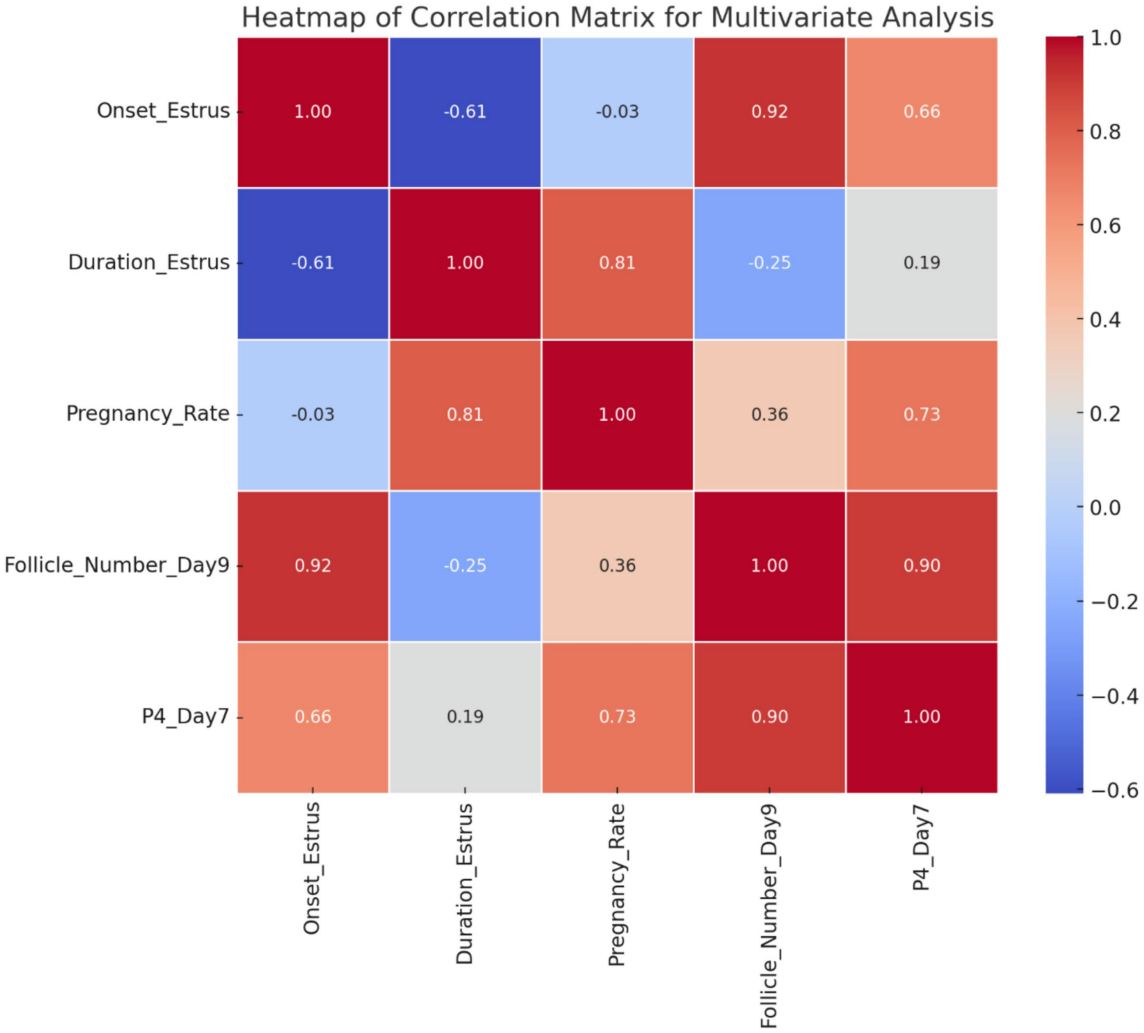
Correlation heatmap of multivariate relationships among estrus, pregnancy rate, follicular number_ Day 9 progesterone levels, Day 7 (G1, G2, G3).

The results demonstrate that Group 1 exhibited the shortest onset of estrus (54.40 ± 4.50 h), while Groups G2 and G3 had significantly delayed onset (71.60 ± 0.51 h and 72.20 ± 4.81 h, respectively). The duration of estrus was similar across groups, with G1 showing a slightly longer duration. Group 2 achieved the highest pregnancy and lambing rates (100%), followed by G1 (80%) and G3 (60%). Interestingly, G3 exhibited the highest twinning rate (66.67%) but lagged in overall reproductive performance. Progesterone levels peaked on day 7, with G2 showing the highest concentration (3.52 ± 0.44 ng/mL), correlating with its superior follicular development. By day 9, G2 displayed the largest follicle number (3.80 ± 0.50) and diameter (0.59 ± 0.02 cm), outperforming G1 and G3 in follicular growth. These findings highlight G2 as the most effective treatment, optimizing estrus induction, reproductive efficiency, and follicular dynamics, as shown in Figure 5. Figure 6 shows the heatmap reveals strong positive correlations between the onset of estrus and follicle number on day 9 (*r* = 0.92) as well as between follicle number on day 9 and progesterone levels on day 7 (*r* = 0.90), suggesting that earlier estrus onset and elevated progesterone levels are associated with greater follicular development. Additionally, a moderate positive correlation is observed between progesterone levels on day 7 and pregnancy rate (*r* = 0.73), indicating the potential role of progesterone in improving reproductive outcomes. Interestingly, the duration of estrus is positively correlated with pregnancy rate (*r* = 0.81) while negatively correlated with the onset of estrus (*r* = −0.61), highlighting that earlier estrus onset may lead to shorter estrus duration but not necessarily reduce reproductive success. These findings underscore the interdependence of hormonal, follicular, and reproductive parameters in treated ewes.

## 4 Discussion

Many estrus synchronization protocols are used in sheep, including intravaginal devices (sponge or CIDR) or injectable hormones for short- or long-term use. This study utilized hormonal injections (progesterone) with a short-term protocol (7 days) modified from protocols by Almadaly et al. (7). Our results indicate that this estrus synchronization protocol is effective during the breeding season. PMSG was administered to the ewes to improve ovulation, resulting in an increased fecundity percentage but not the conception rate (24). This may be due to early embryonic death (25). However, in this study, we observed a high conception rate and fecundity percentage, which may be attributed to the injection of GnRH at the time of breeding. This improves the luteinization of LH-responsive follicles and luteal function, subsequently increasing progesterone concentrations and improving the conception rate, as reported by Hashem and Sallam (26).

PMSG is a gonadotropin commonly used in synchronization and ovulation induction protocols. It functions in the ovary by promoting the expression of angiogenic factors, which leads to follicle neovascularization. This, in turn, increases the ovarian response to LH (27) and improves synchronization outcomes. The present study aimed to establish a nano-drug delivery system (PMSG-loading chitosan nanoparticles; PMSG-ChNPs) to enhance the reproductive performance of short-term progesterone-synchronized ewes by reducing the PMSG dose and achieving quicker synchronization. The success of these goals relies heavily on the physicochemical properties of the formula. The size of nanoparticles (NPs) is crucial for overcoming mucosal tissue barriers and facilitating intracellular uptake (28). Most nanoparticles used in drug delivery systems fall within the size range of 50–250 nm (29).

To the best of our knowledge, our study is unique in determining the effect of PMSG-CsNPs on short-term progesterone estrus synchronization in Ossimi ewes. The findings of this study revealed that the group receiving 600 IU of PMSG exhibited a faster onset of estrus than the groups receiving 300 and 150 PMSG-CsNPs. This may be attributed to the higher dose of PMSG stimulating follicular growth, leading to quicker pituitary endocrine responses, increased follicular activity, higher estrogen levels, and a faster onset of estrus (30). In contrast to other studies, such as McMillan (31), which reviewed the interval to estrus onset in synchronized ewes during the breeding season, ewes synchronized by CIDR with eCG injections of 0, 400, and 800 IU had similar mean intervals to estrus onset (33 vs. 31 vs. 30 h, respectively). This could be due to variations in synchronization protocols, treatment duration, and sheep breeds. There were no significant differences in estrus duration and estrus rate among the groups, indicating that PMSG and PMSG-CsNPs have similarly potent effectiveness in estrus duration and estrus rate.

This study found a higher lambing rate and number of fetuses in the group receiving 300 IU PMSG-CsNPs compared to the other groups. This improvement in PMSG-CsNPs-treated ewes may be due to enhanced pregnancy rates (32) facilitated by the efficient passage of PMSG-CsNPs particles through barriers and cell pores, leading to improved cellular uptake (33). The treatment also supported follicular growth, enhancing the recruitment of small follicles (34). Additionally, the group receiving 300 IU PMSG-CsNPs showed an increase in follicular number and diameters after 72 h of its administration compared to the control group and increased diameters of large follicles on day 9, leading to increased ovulation rates as previously reported (35) and ultimately higher pregnancy rates and fetus numbers, improving fecundity percentage.

Lower doses of PMSG could be adequate to raise estradiol levels by enhancing the growth of antral and non-ovulatory follicles and improving postovulatory luteal function (36). This study demonstrated increased ovarian activity following PMSG injection, as evidenced by a higher number of corpora lutea (CL) 24 h post-injection of PMSG compared to day 0. The rise in CL numbers from day 0 to day 7 of treatment may be attributed to PMSG’s similarity to Luteinizing Hormone (LH), with a longer half-life than LH, leading to luteotropic stimulation in the corpus luteum (37). These effects may involve converting small cells into a larger corpus luteum or enlarging existing luteal cells (38). The study also observed a significant increase in progesterone concentration from day 0 to day 7, possibly due to the rise in the number of extra corpora lutea on the ovarian surface, as reported by Coleson et al. (39) or an increase in the number of luteal cells producing progesterone (40). However, progesterone concentrations decreased in all groups on day 9 compared to day 7, likely due to the luteolytic activity of PGF_2α_ on large CL, as noted by El-Desouky and Hussein (41). Their research indicated that a single dose of PGF_2α_ postpartum caused a sharp decline in serum progesterone levels, followed by increased ovarian activity.

The positive outcomes achieved by utilizing PMSG-CsNPs with the short-term synchronization protocol are likely attributed to the combination of PMSG with chitosan nanoparticles, which are considered to bind to the FSH receptor on the surface of granulosa cells. This results in a 50% reduction in hormonal dosage while maintaining effectiveness and improving stability. This is similar to the findings of Hassanein et al. (42) on GnRH-loaded chitosan nanoparticles. The heatmap shows strong correlations between estrus onset and follicle number (*r* = 0.92), progesterone and follicle number (*r* = 0.90), and a moderate correlation between progesterone and pregnancy rate (*r* = 0.73), highlighting the interdependence of hormones, follicular development, and reproductive outcomes.

Zeta potential is a measurement of surface charge that indicates the stability of particles. High zeta potential values (>30 mV) increase stability and repulsion between particles, preventing them from clumping together. On the other hand, low zeta potential values accelerate particle aggregation (43). Our results indicated that the zeta potential values of PMSG-CsNPs are +11.0, which enhances nanoparticle stability. This is consistent with a study by Danaei et al. (33), which stated that positive zeta potential values, in particular, can enhance cellular uptake and impact cell survival. Due to the simplicity of PMSG-loaded chitosan-TPP nanoparticles and their effectiveness in Ossimi estrus synchronization, this technique can be integrated into various synchronization protocols for farm ewes. The study has some limitations that should be addressed in future research. These include the lack of a detailed assessment of corpus luteum size and small to medium follicle dynamics; postnatal performance parameters such as offspring survival, birth weights, lamb growth rates, and maternal behavior were not evaluated. Although the primary focus was to determine the reproductive efficacy of reduced-dose PMSG-CsNPs protocols, future studies should incorporate these critical production metrics to fully assess the practical farm-level benefits of this approach in sheep breeding systems. Finally, although this study demonstrated biological efficacy, no formal economic comparison between conventional PMSG and the PMSG-CsNPs formulation was performed. Future studies should incorporate cost-effectiveness analyses to determine the practical viability of adopting PMSG-CsNPs in commercial farm settings.

## 5 Conclusion

The innovative approach of short-term progesterone estrus synchronization using PMSG-loaded chitosan-TPP nanoparticles offers promising advancements in reproductive efficiency. By significantly improving pregnancy and lambing rates, this method not only supports the overall productivity of livestock but also contributes to a marked increase in fetus numbers and fecundity percentages. Moreover, the improvement observed in the diameters of large follicles on day six of progesterone synchronization underscores the technique’s effectiveness in optimizing reproductive conditions. These findings highlight a transformative strategy that could reshape breeding practices, ensuring better outcomes for both farmers and their flocks.

## Data Availability

The original contributions presented in the study are included in the article/supplementary material, further inquiries can be directed to the corresponding authors.
